# Treating Young Refugees with a Grief-Focused Group Therapy—A Feasibility Trial

**DOI:** 10.3390/bs15091285

**Published:** 2025-09-19

**Authors:** Anna-Maria Rummel, Anna Vogel, Ruth Rossi, Melanie Jacob, Michael Achtner, Julia Schnauder, Rita Rosner, Hannah Comtesse

**Affiliations:** 1 Department of Psychology, Catholic University Eichstaett-Ingolstadt, 85049 Ingolstadt, Germany; anna-maria.rummel@ku.de (A.-M.R.); melanie.jacob@ku.de (M.J.); rita.rosner@ku.de (R.R.); 2 Schön Klinik Institut für Psychotherapie, 83022 Rosenheim, Germany; rrossi@schoen-klinik.de; 3 Independent Researcher, 85049 Ingolstadt, Germany; michael.achtner@icloud.de; 4 Independent Researcher, 96052 Bamberg, Germany; juliaschnauder@web.de; 5 Department of Psychology, University of Hagen, 58084 Hagen, Germany; hannah.comtesse@fernuni-hagen.de

**Keywords:** PGD, prolonged grief disorder, refugees, treatment, grief therapy, therapy, culturally sensitive, mental health

## Abstract

The death of a loved one has been identified as one of the most commonly reported traumatic experiences among refugees. This phenomenon can lead to the development of prolonged grief disorder (PGD), for which particularly elevated PGD rates were observed among refugees. Currently, there is no treatment specifically designed for refugees with PGD. Therefore, this study investigates the feasibility of a newly developed grief-focused cognitive behavioral group therapy (G-CBT) for this population. Four young refugees aged 16 to 18 and suffering from PGD were assessed pre, during and after treatment. We found a high comorbidity of PGD, posttraumatic stress disorder (PTSD) and depression at baseline. Among completers, clinician-rated PGD symptom severity decreased clinically significantly at posttreatment and remained stable at the three- and six-month follow-ups. The results indicate good feasibility in an outpatient setting with therapists providing positive feedback. However, larger and controlled studies are needed to prove its efficacy.

## 1. Introduction

In 2023, the United Nations High Commissioner for Refugees (UNHCR) reported that 117.3 million people were refugees, asylum-seekers, internally displaced persons, returnees, or stateless due to various reasons, including persecution, conflict, violence, human rights violations, and political conflicts. More than 50 million of them were children (UNHCR, 2024). Although many individuals seek asylum in neighboring countries, a considerable number attempt to reach Europe, often facing dire challenges along the way (UNHCR, 2024). In 2023 there have been over one million first time asylum applications in Europe and more than 300.000 of them applied in Germany, with 30% of applicants in the age range of 16 and 25 years (BAMF, 2024). A notable number of young refugees are unaccompanied. In 2023, the number of unaccompanied minors who submitted applications for asylum in EU countries reached 41.525, marking a 4% increase compared to the previous year. The highest number of reported cases was observed in Germany, with a total of 15.270 cases. Most applications of unaccompanied minors were from Syrians, 33%, or Afghans, 29% (Eurostat, 2024). In addition to their experiences in both their country of origin and during their flight, young refugees face various post-migration challenges in their host country. These include a lack of social support, language barriers, and prolonged asylum application processes. Notwithstanding the aforementioned challenges, often family members remains in their home country, where they frequently lack safety. These challenges have a cumulative effect, resulting in a significant impairment of the mental well-being of those affected (Daniel-Calveras et al., 2022). A Swedish study found that in 2017, unaccompanied young refugees had a suicide rate five times higher than that of the host population. These young refugees had received little or no schooling (67%), endured harassment and abuse (56%), and experienced violence and poverty before seeking asylum in Sweden (67%). Most of them had an unclear asylum situation two years ongoing (Mittendorfer-Rutz et al., 2020).

One of the most frequently reported traumatic experiences for refugees is the death of a family member or friend (Hengst et al., 2018). According to a systematic review, between 28% and 92% of participating refugees reported the death of a loved one (Lechner-Meichsner et al., 2024). A German study of refugees reported that 92% were bereaved, with an average of 5.67 losses (Comtesse & Rosner, 2019). The loss of a loved one is a distressing experience accompanied by emotional suffering and grief. While most individuals experience a decrease in grief symptoms over time, some do not, which can result in prolonged grief disorder (PGD; Lundorff et al., 2017). According to the text revision of the 5th edition of the Diagnostic and Statistical Manual of Mental Disorders (DSM-5-TR), PGD is characterized by yearning for the loved one and persistent thinking about the person. For adolescents, preoccupation may be concentrated on the circumstances surrounding the death. These core symptoms last for at least one year, or six months in adolescents, and are accompanied by emotional distress, loss of relatedness to others and the world, or emotional numbness. These symptoms must exceed cultural and social norms and be associated with functional impairment (APA, 2022). Certain risk factors for PGD have been identified in relation to the circumstances of death (e.g., violent or sudden death, unexpected or multiple losses, exposure to traumatic events) and living conditions after loss (e.g., lack of social support, financial problems; Burr et al., 2024; Heeke et al., 2019; Lobb et al., 2010). These factors often apply to refugees. In particular, extended or ambiguous asylum proceedings have been identified as a risk factor for PGD symptom severity (Comtesse & Rosner, 2019).

The pooled prevalence of PGD in general refugee samples was 33% (Kokou-Kpolou et al., 2020), compared to a prevalence of 13% in non-refugee populations pooled from International Classification of Diseases 11th Revision (ICD 11) and DSM-5-TR estimates (Comtesse et al., 2024). PGD frequently occurs with comorbidities. A meta-analysis revealed that 70% of adult participants experiencing symptoms of PGD also faced another post-loss complication, including depression (63%), anxiety (54%), and PTSD (49%) (Komischke-Konnerup et al., 2021). The co-occurrence of PTSD or depression with PGD has been observed to be high in refugee samples (Lechner-Meichsner et al., 2024; Kokou-Kpolou et al., 2020). A meta-analysis of refugees and asylum seekers reports prevalence rates of 32% for PTSD, 32% for depression and 11% for anxiety disorders (Blackmore et al., 2020). Given the high incidence of post-loss complications observed in young refugees and the documented co-occurrence of PGD with these disorders in adult refugees, it is reasonable to hypothesize that a similar pattern of outcomes is likely to be observed in young refugees. Accordingly, it is reasonable to conclude that young refugees have a high need for mental health support and are at high risk of developing PGD.

To our knowledge, there are currently no grief-focused treatments for (young) refugees. However, there are effectively tested treatment manuals that have been evaluated on other populations samples. A meta-analysis (Hanauer et al., 2024) investigated the efficacy of psychosocial interventions for grief symptoms in children and adolescents. The analysis concluded that uncontrolled therapy studies demonstrated a large effect on grief symptoms at posttreatment. Due to the limited number of controlled studies, no effect size could be calculated nor could a comparison of different types of intervention be made. However, a positive trend in symptom reduction after any kind of treatment was observed. The therapeutic interventions produced large effects in reducing comorbid PTSD symptoms and moderate effects in reducing comorbid depression. None of the included studies examined the young refugee population (Hanauer et al., 2024). A recent meta-analysis (Komischke-Konnerup et al., 2024) of randomized controlled trials (RCT) of grief-focused cognitive behavioral therapy (CBT) for PGD in adults found that CBT interventions had a statistically significant medium effect on reducing PGD symptoms, even compared to an active or competing control condition. This was also true for reduction in PTSD, depression or anxiety symptoms as secondary outcomes. This meta-analysis did not include a refugee sample, either.

Cultural aspects can influence psychotherapy, especially in the context of grief (Aeschlimann et al., 2024). Therefore, interventions with refugees require accommodation for factors such as cultural beliefs, setting, mode, and language. However, only a limited number of studies have examined grief-focused treatments for refugees. A scoping review (Aeschlimann et al., 2024) examined studies that focused on culturally sensitive, grief treatments and support for sociocultural (sub)groups that differ from the majority. The interventions examined in the 18 studies were primarily adapted to different age groups (childhood and adolescence), as well as religious affiliations, sexual orientations, and specific circumstances (e.g., living in a war region, having refugee status). Only two refugee studies were included. The first was a three-day school intervention (guided self-work) compared to a no-intervention control group for young refugees between the ages of 12 and 18 (Kalantari et al., 2012). The other study was a six weeks course of Integrative Adapt Therapy compared to CBT in an individual setting for Myanmar adult refugees (Tay et al., 2020). Both studies were conducted by lay counselors and interventions reduced grief symptoms. A recent study investigated a RCT with 30 bereaved adult Syrian refugees in Switzerland. The intervention consisted of a five-week program that incorporated a newly developed, culturally adapted self-help mobile app. The intervention group exhibited reduced symptoms of PGD following treatment in comparison to a waitlist control group (Aeschlimann et al., 2025). Furthermore, an intensive one-year program consisting of five hours per week at a day clinic was conducted in the Netherlands for adult refugees with persistent complex bereavement disorder (PCBD) and PTSD. This treatment program included individual Brief Eclectic Psychotherapy for Traumatic Grief (BEP-TG) alongside other non-grief specific components. The program was effective for refugees with severe PTSD and PCBD symptoms (De Heus et al., 2017). To our knowledge, however, no study has investigated grief-focused psychotherapeutic treatment for young refugees.

Group therapy is economical and might be preferred by refugees (Lechner-Meichsner & Comtesse, 2022). It is an effective way to combine therapeutic resources, enabling the provision of support to more patients simultaneously. This is particularly true for refugees seeking access to therapeutic services in Germany, where the challenges of accessing psychotherapy are characterized by long wait times (BAfF, 2025). Furthermore, 69% of included group therapies for young refugees in a systematic review have demonstrated significant improvements in mental health symptoms, making it a suitable treatment option (Hutchinson et al., 2022). Given the documented efficacy of CBT in group settings for PGD (Rosner et al., 2011), and the feasibility and effectiveness of group interventions in basic German for young refugees, leading to a significant reduction in symptoms (Pfeiffer et al., 2018), we developed a culturally sensitive, grief-focused CBT group therapy program (G-CBT; Rummel et al., 2023) for young refugees.

The aim of this study was to investigate the feasibility, acceptance, and preliminary efficacy of G-CBT with young refugees in a pilot study. We expected PGD symptoms, PTSD symptoms, and depression symptoms to decrease from the pre- to the posttreatment period and to remain stable over the 3- and 6-month follow-ups.

## 2. Materials and Methods

We conducted an uncontrolled feasibility trial at an outpatient clinic in Germany. The study was approved by the ethics review board of the Catholic University of Eichstätt in May 2023 (Nr. 093-2021). All participants and, in the case of individuals under the age of 18, their legal guardians provided written informed consent. This trial was preregistered in the German Clinical Trials Register, identifier: DRKS00031068. This article adheres to the Structured Assessment of Feasibility (SAFE) reporting guideline (Bird et al., 2014).

### 2.1. Sample and Recruitment

Participants were recruited from March to July 2023 through schools, social workers, counseling centers, child and youth welfare (CYW) facilities, and screening of the outpatient clinic’s therapy requests from the last six months. Study information was also disseminated to psychiatric clinics, doctors, and psychotherapists. The project was also presented at the meeting of the “Migration Forum”, a gathering of people involved with migrants. The study’s aim was to conduct a group of five to eight young refugees. The inclusion criteria were as followed: age between 16 and 21 years; PGD according to DSM-5-TR measured with the Prolonged Grief Disorder 13 Revised scale (PG-13-R; Prigerson et al., 2021); refugee or asylum seeker; sufficient German language skills to follow the initial interview; informed consent provided by the participants and if under 18 years the legal guardian. The presence of severe psychiatric disorders, including acute suicidality or an acute psychotic episode, was an exclusion criterion. Due to recruitment challenges, the final sample comprised four unaccompanied young refugees aged between 16 and 18 years, originating from Afghanistan, Syria, and West Africa, and having already resided in Germany for periods ranging from 6 to 36 months. All of them were currently living in CYW facilities. In Germany, unaccompanied young refugees typically live in residential groups of CYW facilities alongside other youth facing similar challenges. Table 1 contains information regarding the case history of the participants, diagnosed disorders and information on the deceased.

### 2.2. Procedure

**Figure 1:**
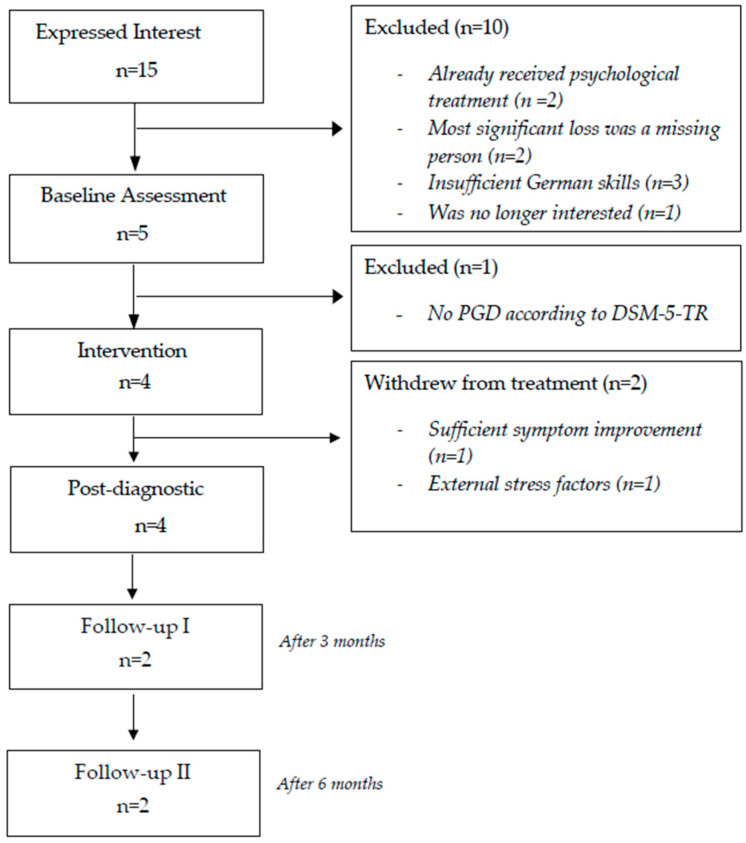
Flowchart of the study.

When social workers or caregivers registered the young people for the study, a brief telephone interview was conducted. Questions were asked about symptoms, knowledge of German, current living circumstances, and information about the loss. In instances where the young people appeared to be eligible, they were invited to participate in an initial interview with a psychotherapist. Caregivers were only present in two cases and only at the beginning and not during the whole interview. The investigation involved the collection of demographic information and details regarding the loss. Furthermore, the presence of any serious psychiatric illnesses, such as acute suicidality, was assessed through structured questions. If none of the exclusion criteria applied and consent to the study criteria was given, a detailed assessment was carried out in one appointment with the young refugees using clinical interviews and questionnaires, with translators if necessary. At the end of the diagnostic process, there was an individual feedback appointment, to which legal guardians or caregivers could also be invited. During this session, the individual’s diagnostic results were explained, and psychoeducation about the identified diagnoses was provided. If participation in G-CBT was indicated, questions about the upcoming group therapy were also clarified. Assessments took place before (pre), after (post), and at 3 (fu-I) and 6 months (fu-II) following treatment. After each session, self-generated questions were recorded to evaluate the course and satisfaction of treatment. Participants received €5 for participating in the post- and follow-up diagnostics. For a detailed flow chart, see Figure 1. The initial interview, the pre-diagnostic and the feedback session were performed by clinical psychologists. Following each session, the participants completed a self-generated questionnaire to evaluate both the treatment and the group. This weekly evaluations along with the post- and follow-up diagnostics were performed by trained clinical raters, who did not administer the treatment. Treatment was administered by two master level therapists who were in advanced postgraduate training for CBT. Furthermore, they received a one-day training course from one of the authors of the G-CBT manual. Following each session, the therapists provided an evaluation of both the session itself and the group. Therapists had weekly supervision sessions led by a supervisor with experience in group therapy for prolonged grief disorder. Adherence was assessed by a trained student. Both the pre-diagnostics and the exposure session were planned with translators, who also acted as a cultural mediator when necessary. The therapeutic intervention was conducted from July to November 2023.

### 2.3. Measures

All measures were applied in German language. Due to a lack of reading skills, all questionnaires and interviews were conducted in the form of interviews. Our approach was based on other studies with refugees (e.g., Comtesse & Rosner, 2019; Rosner et al., 2022).

#### 2.3.1. Primary Outcome

The primary outcome was the Traumatic Grief Inventory—Self Report Plus (TGI-SR+; Lenferink et al., 2022), a valid measurement tool for PGD according to DSM-5-TR and ICD11. It comprises 22 Items with a response scale from 1 (never) to 5 (always). A cut off score of ≥71 indicates possible DSM5-TR and ICD11 PGD cases. Additionally, there are diagnostic rules to determine PGD diagnostic status. An item score of 4 (frequently) or 5 (always) indicates the presence of the requested symptom. In this study, TGI-SR+ total score demonstrated an excellent internal consistency (*α* = 0.95).

#### 2.3.2. Secondary Outcome

The presence of a PGD diagnosis according to DSM-5-TR was confirmed with the PG-13-R (Prigerson et al., 2021). The time criterion for adolescents is a minimum of six months following the loss. For details on diagnosis with the PG-13-R, see Prigerson et al. (2021). We had an excellent internal consistency (*α* = 0.92) in our study.

The presence of comorbid mental disorders according to DSM-5 and ICD-10 was determined using the Diagnostic Short Interview for Mental Disorders (Mini-DIPS; Margraf et al., 2017). It reliably assesses current and lifetime diagnoses and is recommended by the authors from the age of 16.

The occurrence of traumatic events in an individual’s life was assessed using the Life Event Checklist for DSM-5 (LEC-5; Ehring et al., 2014; Weathers et al., 2013). It encompasses a total of 16 distinct categories of traumatic experiences, in addition to an open slot for the inclusion of any event that has not been included in the aforementioned list. Events may be designated as follows: “Happened to me,” “Witnessed it,” “Learned about it,” “Part of my job,” “Not sure,” “Doesn’t apply”.

We used the Posttraumatic Stress Disorder Checklist for DSM-5 (PCL-5; Krüger-Gottschalk et al., 2017), a 20-item questionnaire that utilizes a five-point Likert scale to assess symptoms of PTSD according to DSM-5. This scale ranges from 0 (not at all) to 4 (extremely) and measures symptoms experienced over the previous four weeks. The recommended cut-off score is 33. All items rated as at least 2 (moderately) can be used for the diagnostic rules allowing for a provisional diagnosis according to DSM-5. The total PCL-5-score in our study had an excellent internal consistency (*α* = 0.94).

The Patient Health Questionnaire-9 (PHQ-9; Kroenke et al., 2001) is a widely used reliable instrument to assess depression symptoms. It comprises nine items on a four-point Likert scale ranging from 0 (not at all) to 3 (nearly every day) for the last two weeks. The level of depression severity can be determined in the sum score categories 0–4 (minimal), 5–9 (mild), 10–14 (moderate), 15–19 (moderately severe) and 20–27 (severe). Sum scores of ≥10 were considered as probable major depression. In this study, the sum score showed a good internal consistency (*α* = 0.86).

#### 2.3.3. Adherence

In order to record the therapists’ adherence to the manual, a checklist was created for each session describing the various interventions and dimensions to be addressed in the session. A trained student attended the group sessions and assessed adherence to the manual using this checklist.

Following each session, the therapists completed a brief self-administered questionnaire regarding the therapy session. This publication will only report the results concerning the item dealing with adherence. The following statement was recorded: “In this session, I was able to respond to the needs of my participants without deviating from the manual.” The responses to this question were evaluated using a five-point Likert scale, ranging from “strongly disagree” to “strongly agree.”

#### 2.3.4. Participants Perspective on the Intervention and Adverse Events

In order to monitor adverse events, satisfaction with the treatment, and group dynamics from the participants’ perspective, participants were requested to respond to questions following each session. The items of the questionnaire were composed of both established instruments and self-generated items, which were evaluated by a panel of experts and expressed in simple German. Items whose results are presented in this publication were exclusively self-generated and concerned language comprehension, adverse events, and helpful elements of treatment. In order to prevent socially desirable responses, the young people were encouraged to answer honestly, stating this would be most beneficial to us and there won’t be any negative consequences for them. Language comprehension was determined by the statement “Today I understood the content of the session well” with a binary answer of “yes” or “no”. Participants were then asked to specify in an open text field which factors (e.g., language difficulties, concentration difficulties) contributed to their poor understanding during the session.

Adverse and positive events were documented by the question “Was there a meaningful event in the last week?” with the binary answer option “yes” or “no”. The evaluation of the meaningful event could be classified as positive or negative, and the extent to which it was perceived as stressful and its impact on everyday life could be assessed. Helpfulness of the intervention was rated on a yes/no scale with the question, “Today I found the session helpful”. Participants also provided free text comments about what they found helpful and unhelpful.

### 2.4. Treatment

#### 2.4.1. G-CBT for Refugees

Given the necessity for culturally sensitive treatment manuals and grief-focused therapy for refugees in general, a previously evaluated treatment manual for group therapy for PGD (Rosner et al., 2015), originally designed and examined for an inpatient setting by Rosner et al. (2011), was used as a foundation. However, the newly developed manual took into account an outpatient setting and the specific needs and challenges of refugees. The following key points were considered when developing the manual:

*Post-migration and other stressors:* Since post-migration stressors during treatment are associated with smaller symptom reductions (Djelantik et al., 2020), we addressed these issues as follows, thereby considering the individual most distressing post-migration stressors participants reported in the initial interview. Many refugees’ experience homesickness, which manifests as missing family and friends, ruminating about home and adjustment difficulties and loneliness (Rosner et al., 2022). They still have contact with their families in their home countries, and they have concerns about family members left behind and fears about the asylum process. This could be even more pronounced for young refugees that are unaccompanied, because they are in the host country without their parents or close relatives. Participants could address these concerns or fears during the opening round of each session. Language barriers were identified as a significant stressor. The treatment was conducted in simple German, which provided an additional opportunity to engage with the language in a safe environment. Our participants also reported boredom and loneliness. To address these concerns, we have provided practical tips on how to spend time between sessions, focusing on self-care and emotion regulation. As is the case in other studies on refugees (Genton et al., 2022; Hjern & Kling, 2019), sleep problems were reported. Consequently, we have integrated psychoeducation on good sleep hygiene.

*Skills module:* We integrated psychoeducation about emotions and cultural differences in emotions (e.g., different wording or meaning) to enable participants to speak about their emotions. We also provided skills training to teach participants effective emotional regulation strategies. Furthermore, we discussed the difference between rumination and problem solving. Somatic symptoms are a common symptom of grief in non-Western populations (Hennemann et al., 2023). Therefore, we implemented progressive muscle relaxation (PMR) as a technique for alleviating tension headaches and as a strategy for emotion regulation prior to each session.

*Modifying the exposure:* In order to consider different types of avoidance, we have chosen the approach of the BEP-TG manual (Smid et al., 2015, 2021) which describes four different forms of avoidance in PGD, all of which require a specific form of exposure (see Table 1). We introduced the four avoidance types using illustrative case stories to help participants to identify the most prominent form of avoidance for them. The treatment manual for the therapists defined which specific form of exposure was indicated for which type of avoidance and described the exact procedure. This was implemented in accordance with the BEP-TG manual, with the exception of exposure “in sensu”, which was adopted from the group therapy for PGD (Rosner et al., 2015).

*Cultural aspects and simplification of language:* The worksheets were designed in simple German (BMAS, 2014) and were culturally sensitive (see Supplement S1 for an example). We used keywords and pictograms to make them easier to understand. We minimized the written part; most of the worksheets could be marked with a cross, etc. We integrated culturally specific mourning rituals and highlighted differences among countries, cultures, and religions. Since mourning rituals can be very important for the grieving process (Wojtkowiak et al., 2021; Hilberdink et al., 2023), participants were encouraged and supported in planning their own rituals, whether they were common to their culture or personally meaningful to them. In the end, there was a communal farewell ritual.

#### 2.4.2. G-CBT Treatment Components

G-CBT comprised 11 group sessions of 100 min carried out by two psychotherapists and one individual session à 100 min with one psychotherapist and a translator if requested. A voluntary PMR session of 25 min took place before each 100 min group session from session two. Every group session was structured as follows: (1) An opening round, where participants were invited to report on current mood (by using emotion cards, if desired) or meaningful events of the last week. (2) Discussion of homework exercises. (3) Two to four focus topics per session. (4) Closing round with (self-) reflection. See Table 2 for an overview of the treatment and focus topics.

### 2.5. Statistical Analysis

To evaluate the clinical significance of changes in symptoms related to PGD, PTSD, and depression, the reliable change index (RCI; Jacobson & Truax, 1991) was employed. Symptom change was assessed from pre to post and pre to the two follow-up time points. In addition to the sum value of the respective participant, the test–retest reliability of the measure and the standard deviation (SD) of a control or pretreatment group of a preliminary study or comparative study are required for their calculation (Jacobson & Truax, 1991; Blampied, 2022). The SD should be obtained from a normative sample, or, if it is a specific sample, from a comparative sample (Kendall et al., 1999). The sample selection should be as up-to-date and adequate as possible because it can significantly impact the RCI outcome (Blampied, 2022). Therefore, we focused on refugee samples to ensure the most comparable initial parameters possible. Ideally, all samples would come from the same source. Given the utilization of a normative sample for the purpose of comparison and the absence of studies reporting values for refugees, the TGI-SR+ parameters were derived from a subsample that experienced sudden and violent deaths in a validation study (Kokou-Kpolou et al., 2022). In the case of PCL-5, the test–retest reliability was taken from a German validation study on a clinical sample (Krüger-Gottschalk et al., 2017), while the SD was taken from a refugee sample (Boettcher & Neuner, 2022). We decided to use the SD from a refugee sample here, as this can be reasonable when there are large differences among important participant dimensions (Kendall et al., 1999). However, this meant that two samples were included in the RCI for PCL-5. For the PHQ-9, there was also no refugee sample that had collected both retest reliability and the SD of the entire sample, so we used different studies for each. The test–retest reliability (Wiechers et al., 2023) and the SD (Hornfeck et al., 2023) of the PHQ-9 were both utilized from refugee samples. Thus, a clinically reliable improvement was defined as a reduction of more than 14.09 points in the TGI-SR+ total score, 12.11 points in the PCL-5 and 6.16 points in the PHQ-9.

## 3. Results

### 3.1. Sample Characteristics

The recruitment phase occurred from March to June of 2023. The G-CBT trial started in July 2023. Of the 15 potentially interested participants, 11 had to be excluded prior to baseline diagnostic for various reasons (see flow chart; Figure 1). The final study sample consisted of four unaccompanied young refugees. They were on average 17 years old (range 16–18), all males, and had been in Germany on average for 18.75 months (range 6–36) at the time point of the baseline diagnostic. One participant comes from West Africa, one from Afghanistan, and two from Syria. The young refugees reported, on average, the deaths of three loved ones (range 2–5) and the experience of nine different types of traumatic events (range 7–11). All participants reported experiencing captivity, severe human suffering, fire or explosion, physical assault with a weapon, and sudden violent death. A minimum of two participants disclosed having experienced combat or exposure to a war zone, sudden accidental death, natural disaster, transportation accident, life-threatening illness or injury, and other stressful events not listed.

### 3.2. Outcome Measures

#### 3.2.1. Primary Outcome

**Figure 2:**
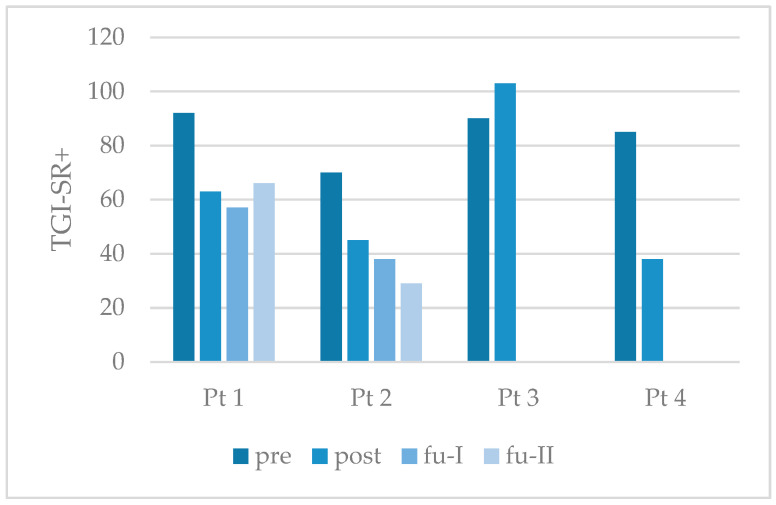
Individual outcome of study participants on symptoms of prolonged grief disorder. Note: TGI-SR+ = Traumatic Grief Inventory-Self Report Plus; Pt = participant; pre = pretreatment; post = posttreatment; fu-I = follow-up after three months; fu-II = follow-up after six months. Bars show the actual total values.

The results showed a reduction in PGD symptoms in three of the four participants (see Figure 2). As shown in Table 3, both completers (Pt 1 and Pt 2) exhibited a clinically significant reduction in symptoms up to six months after treatment. Following the completion of treatment, participants 1, 2, and 4 did not meet the diagnostic criteria for presumably PGD. In the two completers who participated in the follow-up examination, this result remained stable until six months after treatment.

#### 3.2.2. Secondary Outcome

**Figure 3:**
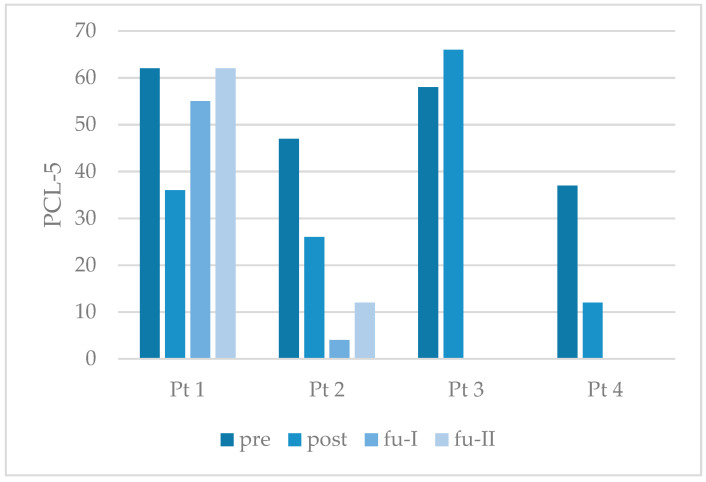
Individual outcome of study participants on symptoms of posttraumatic stress disorder. Note: PCL-5 = Posttraumatic Stress Disorder Checklist for DSM-5; Pt = participant; pre = pretreatment; post = posttreatment; fu-I = follow-up after three months; fu-II = follow-up after six months. Bars show the actual total values.

Following the conclusion of the therapeutic treatment, a reduction in the values of the PCL-5 was observed in both the completers and participant 4 (see Figure 3). These reductions are clinically significant (see Table 3). The participants no longer met the criteria for presumably PTSD. Participant 2 remained stable throughout the follow-up period, in contrast to participant 1, whose improvement declined again. Participant 3 demonstrated a non-clinically significant deterioration.

**Figure 4:**
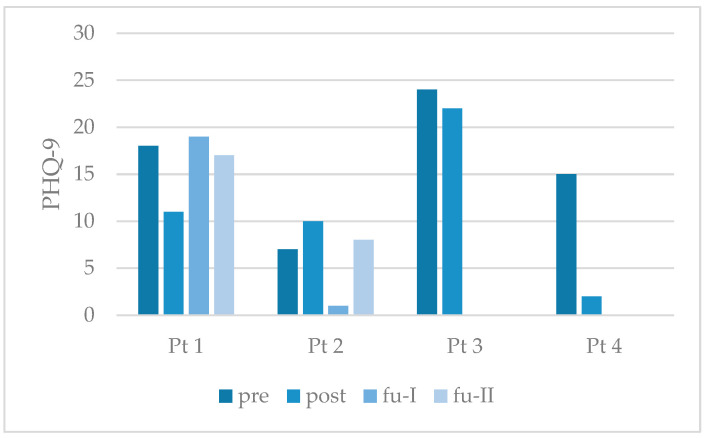
Individual outcome of study participants on symptoms of depression. Note: PHQ-9 = Patient Health Questionnaire-9; Pt = participant; pre = pretreatment; post = posttreatment; fu-I = follow-up after three months; fu-II = follow-up after six months. Bars show the actual total values.

The PHQ-9 scores demonstrated a less substantial reduction, and only reached a clinically significant decrease in participants 1 and 4 at the posttreatment assessment (refer to Figure 4 and Table 3 for details). No consistent decrease in PHQ-9 values was observed over the course of the follow-up period.

### 3.3. Feasibility

#### 3.3.1. Dropout and (Serious) Adverse Events

Of the four participants who initiated the treatment, two participants discontinued the treatment after three or five attended sessions at sessions five and six. The remaining two participants were present for each session. All participants underwent the posttreatment assessment; however, only the participants who completed the treatment participated in the subsequent follow-up assessments. In one case, a participant discontinued the treatment, due to an inability to confront himself with the reality of his loved one’s death. The other participant felt that he had subjectively benefited enough from the treatment.

Adverse events were observed during the therapeutic intervention. The participants were confronted with a number of post-migratory stressors and other stress factors, including the following: “family members being in danger or unreachable,” “destroyed hometown,” “unsafe situation in the home country (fear for family members),” “lack of support with practical life,” “the death of a loved one,” “somatic complaints,” and “insomnia.”. No suicidal crises or other serious adverse events occurred during treatment or the six-month follow-up period.

#### 3.3.2. Participants’ Feedback on the Treatment

After each session, participants were asked to provide feedback on whether they found the session helpful and to identify aspects that were particularly helpful. Three participants rated all of the sessions they attended as helpful. One participant described two sessions as unhelpful; those sessions were associated with adverse events. The following elements and strategies were mentioned as helpful: verbalizing problems, thoughts, and feelings; talking about my own or others’ grief; talking about family; emotion regulation (learn how to deal with feelings such as anger and grief, skills, PMR, etc.); sleep hygiene and cognitive restructuring. Some of the young refugees had little knowledge of the language and were barely at B2 level. According to their own statements, they improved their language skills through treatment and still benefited from the treatment. According to the feedback from participants following the sessions, the participants reported no difficulties with understanding the language during the sessions.

#### 3.3.3. Barriers to Recruitment and Treatment Administration

Despite implementing a comprehensive recruitment strategy, the number of participants in the study was lower than initially planned (5–8 participants). Although social workers indicated that a considerable proportion of refugees under their care exhibited mental health challenges and experiences of losses, only a limited number of individuals contacted us for help. In particular, recruitment did not reach young refugees living with their families, as opposed to those accommodated in CYW facilities.

A series of logistical barriers were encountered, as the four young people originated from different CYW facilities and schools, which made it difficult to find a suitable time slot. However, organizational elements were outsourced to the study management and caregivers of the CYW facilities, thereby allowing therapists and participants to focus on treatment. The commitment of the caregivers seemed essential to initiating and maintaining treatment.

#### 3.3.4. Adherence

The evaluation of the checklists demonstrated that the content of each treatment phase was administered as specified, although there were some slight shifts in content between the individual sessions and in the order within a session. On one occasion, a homework assignment was not given as specified. No interventions were used that were not specified in the manual. In their self-assessments, the therapists stated that they adhered to the manual for nearly every therapy session. There was only one case of partial deviation from the manual during one participant’s individual session due to external factors: the participant had lost contact with his family because his hometown had been destroyed.

## 4. Discussion

Through this study, we attempted to improve the care of young refugees with PGD by developing and testing the feasibility of a grief-focused group therapy. In our study, both PGD and PTSD symptoms were successfully treated with G-CBT. Participants who completed the treatment no longer met the criteria for PGD, and these results remained stable or improved over the following six months. PTSD symptoms were also significantly reduced by grief therapy. Symptoms of depression appeared to be strongly influenced by external factors. Consequently, there are indications that G-CBT could be an effective treatment for young refugees with PGD. The treatment could be carried out adherently, and participants were satisfied with the treatment and able to name several helpful aspects.

The therapeutic intervention was originally developed for young refugees in general. To understand our problems in recruiting accompanied young refugees, we need to consider the common barriers that refugees report in accessing mental health care. A meta-analysis (Dumke et al., 2024) reported seven main barriers. Of these, “understanding of mental health,” “stigma,” and “language barriers,” appear to be the most important in our case. The lack of knowledge about mental illness, the fear of stigmatization, and the culturally different view of illnesses sometimes prevent refugees from even considering psychotherapy (Dumke et al., 2024). For young people living in CYW facilities and being supported by social workers, these barriers may be overcome through education on mental health issues, knowledge about the local psychosocial support network, and assistance with language barriers during the registration process for treatment. Young refugees residing with their parents encounter heightened challenges in this context and are not automatically integrated into support networks, in contrast to unaccompanied refugees. Additionally, the structure of Germany’s social and (mental) healthcare system makes it difficult to provide care for refugees. Refugees are generally not covered by health insurance for the first three years, so emergency treatment is financed by the social welfare system. Consequently, they are less integrated into the healthcare system, making it more difficult to address their needs. Studies have shown a high prevalence of mental health disorders among refugees (Patanè et al., 2022), yet many do not receive treatment (BAfF, 2025). Our study also revealed this issue: although counseling centers and social workers reported the needs of young refugees, no one from outside the CYW facility approached us. However, psychotherapy may not always be the best option, and in some cases, those affected are not prepared to engage in it. Therefore, implementing a case management system that adequately assesses needs and provides concrete, and if needed, acute support would be of importance. Political restructuring is necessary to facilitate access and provide all young refugees with adequate healthcare. Of the 15 inquiries reviewed, none were from accompanied young refugees. Mental health studies in general have also documented challenges in recruiting young people (Djelantik et al., 2024). A scope review indicates that recruitment via social media can yield more successful outcomes than conventional methods (Smith et al., 2023). For subsequent studies, the implementation of social media recruitment in multiple languages to address participants in their native language may prove beneficial.

However, due to our recruitment limited of unaccompanied refugees, we adapted the therapeutic program to address the specific challenges they face. During the opening round of each session, participants could discuss matters related to contact with parents living far away, the home country and circumstances there, and challenges encountered in the CYW facilities with the therapists. Caregivers from the CYW facilities were only integrated during the diagnostic feedback and in between for organizational purposes in order to ensure reliability of participation in treatment. Since our participants were at least 16 years old and the treatment took place in a group setting, we decided against including caregivers in therapeutic sessions. One of the main barriers to mental health care for refugees is the “language barrier” (Dumke et al., 2024). Despite the participants’ limited language skills, the intervention was conducted in simple German due to the group setting. Simplifying the content in conjunction with the use of visual aids, such as pictograms and keywords, proved to be an effective strategy in our study. Despite their limited language skills, the participants reported that they understood the content well and were able to benefit from the intervention. Consequently, despite the absence of language mediators in the group sessions, this barrier was effectively reduced and the participants had the opportunity to improve their German language skills in a controlled setting.

Despite their young age, all of the included participants reported multiple losses of loved ones and generally experienced high levels of symptomatic and daily burden. In the present study, all participants met criteria for PTSD and depression in addition to PGD, as indicated by the diagnostic interview. Additionally, all had sleep disturbances and some suicidal ideation. The sample shows evidence of significant psychological distress, which is consistent with the findings of other studies reporting increased levels of comorbidities with PGD, particularly in refugee samples (Kokou-Kpolou et al., 2020).

A reduction in PGD symptom severity from pre- to post-measurement was observed in all but one participant. However, it should be noted that not all participants demonstrated a clinically significant change. During subsequent follow-up evaluations, a sustained enhancement or an additional decrease in the symptom scores was observed in the two completers. Following a comprehensive evaluation, it was determined that they no longer met the PGD criteria. A pre-post comparison of PTSD symptoms reveals a similar outcome; three participants no longer fulfilled the criteria for PTSD. Although participant 1 exhibited a clinically significant improvement in PTSD symptoms at posttreatment, these improvements were reversed during the follow-up period, with the occurrence of a new traumatic event as a possible explanation. Participant 2 demonstrated a significant decrease in PTSD symptoms, which were consistent during the follow-up period. This observation can potentially be attributed to the overlap between the index event of PTSD and the loss. In this case, the procedure of exposure (“in sensu”) was similar to that used in evidence-based PTSD therapies.

The depressive symptoms showed a diverse picture, potentially associated with particular situations or life circumstances. Participants 1 and 4 had their asylum applications approved at post-diagnostic and demonstrated clinically significant improvements in the RCI. In comparison, participant 2 exhibited fluctuations in symptom severity that were not classified as clinically significant changes. We hypothesized that these fluctuations could be influenced by external factors, such as the loss of contact with his mother at posttreatment. This adverse event might potentially exert a more substantial influence on the depressive symptoms observed in comparison to those associated with PGD and PTSD at posttreatment. Overall, depression did not appear to be significantly influenced by the treatment, but rather by external circumstances and incidents. These are individual cases, so no causal conclusion can be drawn about the refugee population. However, our observations align with the literature, as post-migration stressors are associated with an increased risk of depression (Nutsch & Bozorgmehr, 2020).

Two participants discontinued treatment, one due to enough improvement and the other due to severe grief-specific avoidance. Participant 3 exhibited a non-clinically reliable deterioration in PGD and PTSD symptoms following the withdrawal of treatment. However, the participant withdrew from treatment at his own request, due to an inability to confront himself with the fact of his loved one’s death. Avoidance behavior is associated with the maintenance of symptoms in PGD (Boelen et al., 2007). In addition to the pronounced avoidance behavior, participant 3 also reported the presence of numerous post-migration stressors, which is why treatment is perceived as too stressful at the present time. Our therapeutic method does not appear to be appropriate in his situation and an alternative approach, such as a focus on problem solving as in PCT, may be more suitable. PCT presents an alternative approach to managing avoidance behavior by addressing current problems (Vogel et al., 2021). Participant 4 demonstrated a clinically significant reduction in symptoms of PGD, PTSD and depression after a relatively brief treatment period. This development seemed to be associated with the positive asylum decision and the approval of the family reunification, which ultimately led to the intended discontinuation of treatment. These factors may have contributed to a reduction in the stressors he had previously indicated, including fear of being sent back to his country of origin, feelings of isolation, and concern for the well-being of his family. Due to the participant’s non-availability for follow-up diagnostics, the stability of the observed symptom reduction remains unclear. Consequently, the actual dropout rate is 25%, which is consistent with the norm observed in the field of refugee studies. Rates ranging from 0 to 64% have been documented in single efficacy studies of refugees (Semmlinger & Ehring, 2021). Given the number of participants who discontinued treatment, the assessment of readiness to change should be questioned and, in future, assessed early on in the screening process. Due to the frequently challenging living conditions experienced by refugees, as well as the specific challenges they face measuring readiness and motivation, could serve as an effective approach to better channeling the type of support indicated and its prioritization.

Therapists followed the manual closely. Only minor deviations from structure in two points were made. However, it became apparent that adherence benefited from the small number of participants in some sessions. With a larger number of participants, time management might be more challenging. Therefore, G-CBT should be examined with larger sample sizes to verify the feasibility in terms of time. In one session, a therapist declared that it was a challenge for him to adhere to the manual. In this case, the external circumstances must be considered. At this time point, the participant’s hometown had been destroyed, and he was not able to reach his family. Consequently, a conversation was held before the exposure, followed by a shorter, less intensive exposure due to his situation. This made it more difficult for the participant to engage with the exposure and for the therapist to follow the manual. This shows that this clientele requires additional optional meetings to respond flexibly to adverse events and other stressors.

Our study has some limitations. First, our sample was small and purely male. However, it is representative of the distribution by country of origin of URY in Germany (Eurostat, 2024), yet it is not possible to make a statement about the effectiveness of the treatment or the generalizability of the results. Furthermore, the study was conducted to test the feasibility. Due to its uncontrolled design, statements about the effectiveness of the treatment are limited. We cannot clearly say whether the treatment itself contributed to the improvement, or if factors such as being part of a group and having a supportive connection affected symptoms. Another limitation is the large number of external factors and post-migration stress factors that occurred during treatment. This complicates the evaluation of the intervention’s efficacy, and its feasibility can only be determined to a limited extent. Nevertheless, the study has a high external validity, as young refugees are confronted with many stressors and problems in everyday life. Other studies have reported on post-migration stressors, such as an uncertain visa status, issues with school or housing, and language problems, which impact mental health (Daniel-Calveras et al., 2022; Nickerson et al., 2019). No standardized language level was set as an inclusion criterion prior to treatment. Participants only needed to be able to follow the initial interview. Furthermore, some questionnaires used in this study are not validated with refugees in general, particularly young refugees. The validity of these instruments is therefore questionable, especially when they are used with interpreters, as was the case with the pretreatment diagnostics. Finally, understanding of the content of each session can only be assessed to a limited extent, as social desirability may have played a role in the responses despite our efforts to counteract this.

Despite the study’s limitations, it offers unique strengths. To the best of our knowledge, this is the first study to test grief-focused group therapy for young refugees with PGD. The efficacy of the therapeutic intervention was observed, despite the presence of external stressors and limited German language skills, particularly among those who completed the treatment program. The external validity of the study is good, as it includes systematic documentation of stressors and other relevant issues on a weekly basis. On the one hand, this reveals the impact of these factors on the treatment and shows which adversities young refugees are exposed to. The evaluation of all disorder-specific symptoms was conducted through clinical interviews at all measurement time points.

This is the first study with this vulnerable sample that shows that group therapy is feasible. A pragmatic adaptation of the evidence-based content, including the language, does not appear to negatively affect effectiveness, which is very encouraging for working with the group of (young) refugees. The next step in this research is to conduct a more thorough investigation of the intervention’s effectiveness using RCTs. Furthermore, it is essential to expand the targeted age group and consider the unique challenges of each age group. In practice, paying attention to post-migration stressors has been shown to be of great importance for treatment compliance and access to treatment. Therefore, close cooperation with social workers and explicit optional sessions that address these stressors should be considered. In general, close networking with CYW facilities seems sensible, and the offer of an outreach group program should also be investigated in future studies.

## 5. Conclusions

In summary, despite the limitations, the treatment successfully treated both PGD and PTSD symptoms. Participants who completed the treatment no longer met the criteria for PGD, and these results remained stable or improved over the following six months. In most cases, the symptoms of PTSD were also significantly reduced by grief therapy. Symptoms of depression appeared to be strongly influenced by external factors. Consequently, there are indications that G-CBT could be an effective treatment for young refugees with PGD. The treatment was carried out adherently, without any treatment-related adverse events. Despite their high symptom burden, difficult living conditions, and post-migration factors, most participants rated the treatment as helpful. Nevertheless, the difficulty of recruiting has shown that refugees need better access to support services, and that support services need better access to those affected. Until then, we hope this study will serve as a basis for further research, particularly randomized controlled trials examining the effectiveness of grief-related therapy for (young) refugees and the necessary adjustments.

## Figures and Tables

**Table 1:** Overview of Participants.

Participant	Brief Case History	Categorical Diagnosis	Deceased/Significant Loss
1	The young refugee is living in a CYW facility. He reports several deaths and mentions five by name, all unnatural deaths either by murder or accident; he has contact with his family in his home country. He went to school in Germany and his asylum application was approved at the end of treatment.	PGD Major depression PTSD Current and lifetime no suicidal ideation;	Relative Murdered Unexpected death 8 months since the loss
2	The young refugee is living in a CYW facility. He reports several deaths and mentions two by name, one from illness and one from war; he has contact with his family in his home country. At the beginning of treatment, he started with school, and he applied for asylum.	PGD Major depression PTSD No suicidal ideation; death wishes in the past.	Friend Death by a bomb Unexpected death 36 months since the loss
3	The young refugee is living in a CYW facility. He reports two deaths, both unexpected but natural; he has no family left. He was in vocational school and has residency permits.	PGD Major depression PTSD Participant had suicidal thoughts before treatment but credibly distanced himself from suicidal thoughts and attempts during treatment.	Parent Death by illness Unexpected death 21 months since the loss
4	The young refugee is living in a CYW facility. He reports two deaths, one from illness and one from war—he was present at both deaths. At the beginning of treatment, he went to school. His asylum application was approved during the treatment.	PGD Major depression PTSD Self-harm and suicidal thoughts in the past, currently credibly distanced	Relative Death by illness Unexpected death 12 months since the loss

**Table 2:** G-CBT Treatment Components.

Treatment Phase (Session Numbers)	Focus Topics	Treatment Strategies	
Introduction and psychoeducation (1–3)	Building motivation	Psychoeducation on psychotherapy; group rules; personal therapy goals	
	Characteristics and consequences of my grieving process	Reflection on the participants’ own grieving process; common mid- and long-term consequences of grieving	
	Cultural differences in grieving	Cultural variations in mourning practices and rituals; open non-judgmental reflection on traditions, temporal aspects, attire, religious belief, etc., regarding grief.	
	Development of PGD	Difference between integrated and prolonged grief using an adapted and simplified version of the grief model according to Znoj (2004).	
	Symptoms and coping	Coping attempts to deal with the current situation; symptoms as dysfunctional coping attempts.	
	Sleep hygiene	Introduction of sleep hygiene strategies.	
Emotions (4–5)	Psychoeducation on emotions	Introduction of the main emotions and emotions’ functions (e.g., providing information about the situation, expression, messages, etc.).	
	Emotion regulation and skills	Suppression of emotions and problem behavior; emotion regulation; theoretical information and practical use of skills according to DBT (Bohus & Wolf-Ahrehult, 2018).	
	Rumination and problem solving	Difference between rumination and problem solving; (dys-)functionality of rumination in PGD.	
	Strategies to deal with rumination behavior	Anti-rumination strategies such as “rumination stop”, “best friends trick”, “worry time”, “conscious memorial time”, “radical acceptance”.	
Avoidance and exposure (6–9)	Avoidance in PGD (Avoidance types)	Psychoeducation on avoidance in PGD; introducing four types of avoidance: “avoiding the reality of loss/emotions”, “avoiding certain situations or objects”, “avoiding memories of certain events” and “holding on to grief behavior” with example stories (BEP-TG; Smid et al., 2015, 2021).	
	Functionality of avoidance	Functionality of the introduced PGD avoidance types; reflection on one’s own avoidance behavior and functionality of it.	
	Reducing avoidance behavior	Approaches to reduce avoidance, depending on type.	
	Presentation of the deceased person	Participants introduce their deceased person and what made him/her special to them by presenting pictures, stories, collages etc.	
	Exposure (Individual Session)	Individual approach depends on the most dominant avoidance type.	
		**Avoidance type**	**Exposure**
		Avoiding the reality of loss/emotions	Allowing grief (recall memories)
		Avoiding certain situations or objects	In vivo (confrontation with situations/objects)
		Avoiding memories of certain events	In sensu (confrontation with the worst moment)
		Holding on to grief behavior	Reduction in grief behavior (reflection of the grieving behavior; planning the grieving process); building alternative behaviors.
Cognitive restructuring (10–11)	Psychoeducation on dysfunctional thoughts	Psychoeducation on dysfunctional thoughts and the relationship between thoughts, emotions and behavior; introduction to the ABC scheme based on Ellis (1991).	
	Restructuring	Restructuring of grief related dysfunctional thoughts in the group; individual restructuring with worksheets; discussing different restructuring possibilities in the group	
	My plan for difficult times	Planning for difficult upcoming events such as birthdays, holidays etc.	
Conclusion (12)	My future	Changes due to treatment; experiences of growth; imagine the future.	
	Importance of rituals	Diversity of rituals; participants reflect on advantages of individual grieving rituals; finding an individual grieving ritual for each participant.	
	Collective grieving ritual	The group closes with a collective grieving ritual to say farewell to the deceased.	

**Table 3:** Reliable change in individual outcome of study participants.

		Difference in Total Values		
Participant	Outcome	Pre-Post	Pre-fu-I	Pre-fu-II
1	TGI-SR+	29 *	35 *	26 *
	PCL-5	26 *	7	0
	PHQ-9	7 *	−1	1
2	TGI-SR+	25 *	32 *	41 *
	PCL-5	21 *	43 *	35 *
	PHQ-9	−3	6	−1
3	TGI-SR+	−13		
	PCL-5	−8		
	PHQ-9	2		
4	TGI-SR+	47 *		
	PCL-5	25 *		
	PHQ-9	13 *		

Note. TGI-SR+ = Traumatic Grief Inventory-Self Report Plus; PCL-5 = Posttraumatic Stress Disorder Checklist for DSM-5; PHQ-9 = Patient Health Questionnaire-9; pre-post = improvement from pretreatment to posttreatment; pre-fu-I = improvement from pretreatment to follow-up after three months; Pre-fu-II = improvement from pretreatment to follow-up after six months. * = Clinically reliable change—RCI > 1.96 (Jacobson & Truax, 1991).

## Data Availability

Data are not available due to privacy and ethical restrictions. G-CBT manual is available on request from the first author.

## Supplementary Materials

The following supporting information can be downloaded at: https://www.mdpi.com/article/10.3390/bs15091285/s1.

## Abbreviations

The following abbreviations are used in this manuscript:

PGD	Prolonged Grief Disorder
G-CBT	Grief-focused Cognitive Behavioral Therapy
DSM-5-TR	Diagnostic and Statistical Manual of Mental Disorders, 5th Edition, Text Revision
ICD-10/11	International Classification of Diseases, 10th/11th Revision
PTSD	Posttraumatic Stress Disorder
UNHCR	United Nations High Commissioner for Refugees
CYW	Child and Youth Welfare
RCT	Randomized Controlled Trial
CBT	Cognitive Behavioral Therapy
PCBD	Persistent Complex Bereavement Disorder
BEP-TG	Brief Eclectic Psychotherapy for Traumatic Grief
PG-13-R	Prolonged Grief Disorder 13 Revised Scale
Pre	Before the treatment
Post	After the treatment
Fu-I	Three months after the treatment
Fu-II	Six months after the treatment
TGI-SR+	Traumatic Grief Inventory Self Report Plus
Mini-DIPS	Diagnostic Short Interview of Mental Disorder
LEC-5	Life Event Checklist for DSM-5
PCL-5	Posttraumatic Stress Disorder Checklist for DSM-5
PHQ-9	Patient Health Questionnaire-9
PMR	Progressive Muscle Relaxation
RCI	Reliable Change Index
SD	Standard Deviation

## References

[B1-behavsci-15-01285] Aeschlimann A., Heim E., Killikelly C., Arafa M., Mearcker A. (2024). Culturally sensitive grief treatment and support: A scoping review. SSM Mental Health.

[B2-behavsci-15-01285] Aeschlimann A., Heim E., Killikelly C., Mahmoud N., Haji F., Stoeckli R. T., Aebersold M., Thoma M., Maercker A. (2025). Cultural adaption of a self-help app for grieving Syrian refugees in Switzerland. A feasibility and acceptability pilot-RCT. Internet Interventions.

[B3-behavsci-15-01285] APA—American Psychiatric Association (2022). Diagnostic and statistical manual of mental disorders—Text revision.

[B4-behavsci-15-01285] BAfF—Bundesweite Arbeitsgemeinschaft für psychosoziale Zentren für Flüchtlinge und Folteropfer (2025). Flucht & gewalt: Psychosozialer versorgungsbericht deutschland 2025, fokus grenzgewalt.

[B5-behavsci-15-01285] BAMF—Bundesamt für Migration und Flüchtlinge (2024). Das bundesamt in zahlen 2023. Asyl, migration und integration.

[B6-behavsci-15-01285] Bird V., Le Boutillier C., Leamy M., Williams J., Bradstreet S., Slade M. (2014). Evaluating the feasibility of complex interventions in mental health services: Standardised measure and reporting guidelines. British Journal of Psychiatry.

[B7-behavsci-15-01285] Blackmore R., Gray K. M., Boyle J. A., Fazel M., Ranasinha S., Fitzgerald G., Misso M., Gibson-Helm M. (2020). Systematic review and meta-analysis: The prevalence of mental illness in child and adolescent refugees and asylum seekers. Journal of the American Academy of Child & Adolescent Psychiatry.

[B8-behavsci-15-01285] Blampied N. M. (2022). Reliable change and the reliable change index: Still useful after all these years?. The Cognitive Behavior Therapist.

[B9-behavsci-15-01285] BMAS—Bundesministerium für Arbeit und Soziales (2014). Leichte sprache. Ein ratgeber.

[B10-behavsci-15-01285] Boelen P. A., de Keijser J., van den Hout M. A., van den Bout J. (2007). Treatment of complicated grief: A comparison between cognitive-behavioral therapy and supportive counseling. Journal of Consulting and Clinical Psychology.

[B11-behavsci-15-01285] Boettcher V. S., Neuner F. (2022). The impact of an insecure asylum status on mental health of adult refugees in Germany. Clinical Psychology in Europe.

[B12-behavsci-15-01285] Bohus M., Wolf-Ahrehult M. (2018). Interaktives skillstraining für borderline-patienten. Das therapeutenmanual.

[B13-behavsci-15-01285] Burr C., Zachariae R., Komischke-Konnerup K. B., Marello M. M., Schierff L. H., O’Connor M. (2024). Risk factors for prolonged grief symptoms: A systematic review and meta-analysis. Clinical Psychology Review.

[B14-behavsci-15-01285] Comtesse H., Rosner R. (2019). Prolonged grief disorder among asylum seekers in Germany: The influence of losses and residence status. European Journal of Psychotraumatology.

[B15-behavsci-15-01285] Comtesse H., Smid G. E., Rummel A.-M., Spreeuwenberg P., Lundorff M., Dückers M. L. A. (2024). Cross-national analysis of the prevalence of prolonged grief disorder. Journal of Affective Disorders.

[B16-behavsci-15-01285] Daniel-Calveras A., Baldqaui N., Baeza I. (2022). Mental health of unaccompanied refugee minors in Europe: A systematic review. Child Abuse & Neglect.

[B17-behavsci-15-01285] De Heus A., Hengst S. M., de la Rie S. M., Djelantik A. A. A. M. J., Boelen P. A., Smid G. E. (2017). Day patient treatment for traumatic grief: Preliminary evaluation of a one-year treatment programme for patients with multiple and traumatic losses. European Journal of Psychotraumatology.

[B18-behavsci-15-01285] Djelantik A. A. A. M. J., deHeus A., Kuiper D., Kleber R. J., Boelen P. A., Smid G. E. (2020). Post-migration stressors and their association with symptom reduction and non-completion during treatment for traumatic grief in refugees. Frontier in Psychiatry.

[B19-behavsci-15-01285] Djelantik A. A. A. M. J., van Es C. M., Lahuis A. M., Mooren N. (2024). The challenges of conducting mental health research among resettled refugee populations: An ecological framework from a researchers’ perspective. International Journal of Mental Health.

[B20-behavsci-15-01285] Dumke L., Wilker S., Hecker T., Neuner F. (2024). Barriers to accessing mental health care for refugees and asylum seekers in high-income countries: A scope review of reviews mapping demand and supply-side factors onto a conceptual framework. Clincal Psychology Review.

[B21-behavsci-15-01285] Ehring T., Knaevelsrud C., Krüger A., Schäfer I. (2014). German version of the life events checklist for DSM-5 (LEC-5) and the PTSD checklist for DSM-5 (PCL-5).

[B22-behavsci-15-01285] Ellis A. (1991). The revised ABC’s of rational-emotive therapy (RET). Journal of Rational-Emotive & Cognitive-Behavior Therapy 9(3).

[B23-behavsci-15-01285] Eurostat (2024). Asylum applications—Annual statistics. statistics explained.

[B24-behavsci-15-01285] Genton P. C., Wang J., Bodenmann P., Ambresin A.-E. (2022). Clinical profile and care pathways among unaccompanied minors asylum seekers in Vaud, Switzerland. International Journal of Adolescent Medicine and Health.

[B25-behavsci-15-01285] Hanauer C., Telaar B., Rosner R., Doering B. K. (2024). The efficacy of psychosocial interventions for grief symptoms in bereaved children and adolescents: A systematic review and meta-analysis. Journal of Affective Disorders.

[B26-behavsci-15-01285] Heeke C., Kampisiou C., Niemeyer H., Knaevelsrud C. (2019). A systematic review and meta-analysis of correlates of prolonged grief disorder in adults exposed to violent loss. European Journal of Psychotraumatol.

[B27-behavsci-15-01285] Hengst S. M. C., Smid G. E., Laban C. J. (2018). The effects of traumatic und multiple loss in psychotraumathology, disability, and quality of life in Iraqi asylum seekers in the Netherlands. The Journal of Nervous and Mental Disease.

[B28-behavsci-15-01285] Hennemann S., Killikelly C., Hyland P., Maercker A., Witthöft M. (2023). Somatic symptom distress and ICD-11 prolonged grief in large intercultural sample. European Journal of Psychotraumatology.

[B29-behavsci-15-01285] Hilberdink C. E., Ghainder K., Dubanchet A., Hinton D., Djelantik A. A. A. M. J., Hall B. J., Bui E. (2023). Breavement issues and prolonged grief disorder: A global perspective. Ambridge Prisms: Global Mental Health.

[B30-behavsci-15-01285] Hjern A., Kling S. (2019). Health care needs in school-age refugee children. International Journal of Environmental Research and Public Health.

[B31-behavsci-15-01285] Hornfeck F., Eglinsky J., Garbade M., Rosner R., Kindler H., Pfeiffer E., Sachser C. (2023). Mental health problems in unaccompanied young refugees and the impact of post-flight factors on PTSS, depression and anxiety—A secondary analysis of the Better Care study. Frontiers in Psychology.

[B32-behavsci-15-01285] Hutchinson R., King N., Majumder P. (2022). How effective is group intervention in the treatment for unaccompanied and accompanied refugee minors with mental health difficulties: A systematic review. International Journal of Social Psychiatry.

[B33-behavsci-15-01285] Jacobson N. S., Truax P. (1991). Clinical significance: A statistical approach to defining meaningful change in psychotherapy research. Journal of Consulting and Clinical Psychology.

[B34-behavsci-15-01285] Kalantari M., Yule W., Dyregrov A., Neshatdoost H. A., Ahmadi S. J. (2012). Efficacy of writing for recovery on traumatic grief symptoms of afghani refugee bereaved adolescents: A randomized control trial. OMEGA-Journal of Death and Dying.

[B35-behavsci-15-01285] Kendall P. C., Marrs-Garcia A., Nath S. R., Sheldrick R. C. (1999). Normative comparisons for the evaluation of clinical significance. Journal of Consulting and Clinical Psychology.

[B36-behavsci-15-01285] Kokou-Kpolou C. K., Lenferink L. I. M., Brunnet A. E., Park S., Megalakaki O., Boelen P., Cénat J. M. (2022). The ICD-11 and DSM-5-TR prolonged grief criteria: Validation of the Traumatic Grief Inventory-Self Report Plus using exploratory factor analysis and item response theory. Clinical Psychology and Psychotherapy.

[B37-behavsci-15-01285] Kokou-Kpolou C. K., Moukouta C. S., Masson J., Bernoussi A., Cénat J. M., Bacqué M. F. (2020). Correlates of grief-related disorders and mental health outcomes among adult refugees exposed to trauma and bereavement: A systematic review and future research directions. Journal of Affective Disorders.

[B38-behavsci-15-01285] Komischke-Konnerup K. B., Zachariae R., Boelen P. A., Marello M. M., O’Connor M. (2024). Grief-focused cognitive behavioral therapies for prolonged grief symptoms: A systematic review and meta-analysis. Journal of Consulting and Clinical Psychology.

[B39-behavsci-15-01285] Komischke-Konnerup K. B., Zachariae R., Johannsen M., Nielsen L. D., O’Connor M. (2021). Co-occurrence of prolonged grief symptoms and symptoms of depression, anxiety, and posttraumatic stress in bereaved adults: A systematic review and meta-analysis. Journal of Affective Disorder Report.

[B40-behavsci-15-01285] Kroenke K., Spitzer R. L., Williams J. B. W. (2001). The PHQ-9. Validity of a brief depression severity measure. Journal of General Internal Medicine.

[B41-behavsci-15-01285] Krüger-Gottschalk A., Knaevelsrud C., Rau H., Dyer A., Schäfer I., Schellong J., Ehring T. (2017). The German version of the Posttraumatic Stress Disorder Checklist for DSM-5 (PCL-5): Psychometric properties and diagnostic utility. BMC Psychiatry.

[B42-behavsci-15-01285] Lechner-Meichsner F., Comtesse H. (2022). Beliefs about causes and cures of prolonged grief disorder among Arab and Sub-Saharan African refugees. Frontiers in Psychiatry.

[B43-behavsci-15-01285] Lechner-Meichsner F., Comtesse H., Olk M. (2024). Prevalence, comorbidities, and factors associated with prolonged grief disorder, posttraumatic stress disorder and complex posttraumatic stress disorder in refugees: A systematic review. Conflict and Health.

[B44-behavsci-15-01285] Lenferink L. I. M., Eisma M. C., Smid G. E., de Keijser J., Boelen P. A. (2022). Valid measurement of DSM-5 persistent complex bereavement disorder and DSM-5-TR and ICD-11 prolonged grief disorder: The Traumatic Grief Inventory-Self Report Plus (TGI-SR+). Comprehensive Psychiatry.

[B45-behavsci-15-01285] Lobb E. A., Kristjanson L. J., Aoun S. M., Monterosso L., Halkett G. K. B., Davies A. (2010). Predictors of complicated grief: A systematic review of empirical studies. Death Studies.

[B46-behavsci-15-01285] Lundorff M., Holmgren H., Zachariae R., Farver-Vestergaard I., O’Connor M. (2017). Prevalence of prolonged grief disorder in adult bereavement: A systematic review and meta-analysis. Journal of Affective Disorders.

[B47-behavsci-15-01285] Margraf J., Cwik J. C., Pflug V., Schneider S. (2017). Strukturierte klinische Interviews zur Erfassung psychischer Störungen über die Lebensspanne. Zeitschrift für Klinische Psychologie und Psychotherapie.

[B48-behavsci-15-01285] Mittendorfer-Rutz E., Hagström A., Hollander A. C. (2020). High suicide rates among unaccompanied minors/youth seeking asylum in sweden. Crisis.

[B49-behavsci-15-01285] Nickerson A., Byrow Y., O’Donnell M., Mau V., McMahon T., Pajak R., Li S., Hamiltion A., Minihan S., Liu C., Bryant R. A., Berle D., Liddell B. J. (2019). The association between visa insecurity and mental health, disability and social engagement in refugees living in Australia. European Journal of Psychotraumatology.

[B50-behavsci-15-01285] Nutsch N., Bozorgmehr K. (2020). Der Einfluss postmigratorischer Stressoren auf die Prävalenz depressiver Symptome bei Geflüchteten in Deutschland. Analyse anhand der IAB-BAMF-SOEP-Befragung 2016. Bundesgesundheitsblatt—Gesundheitsforschung—Gesundheitsschutz.

[B51-behavsci-15-01285] Patanè M., Ghane S., Karyotaki E., Cuijpers P., Schoonmade L., Tarsitani L., Sijbrandij M. (2022). Prevalence of mental disorders in refugees and asylum seekers: A systematic review and meta-analysis. Cambridge Prisms: Global Mental Health.

[B52-behavsci-15-01285] Pfeiffer E., Sachser C., Rohlmann F., Goldbeck L. (2018). Effectiveness of a trauma-focused group intervention for young refugees: A randomized controlled trial. Journal of Child Psychology and Psychiatry, and Allied Disciplines.

[B53-behavsci-15-01285] Prigerson H. G., Boelen P. A., Xu J., Smith K. V., Maciejewski P. K. (2021). Validation of the new DSM-5-TR criteria for prolonged grief and the PG-13-Revised (PG-13-R) scale. World Psychiatry.

[B54-behavsci-15-01285] Rosner R., Hagl M., Bücheler L., Comtesse H. (2022). Homesickness in asylum seekers: The role of mental health and migration-related factors. Frontiers in Psychiatry.

[B55-behavsci-15-01285] Rosner R., Lumbeck G., Geissner E. (2011). Effectiveness of an inpatient group therapy for comorbid complicated grief disorder. Psychotherapy Research.

[B56-behavsci-15-01285] Rosner R., Pfoh G., Rojas R., Brandstätter M., Rossi R., Lumbeck G., Kotoučová M., Hagl M., Geissner E. (2015). Anhaltende trauerstörung. Manuale für die einzel- und gruppentherapie.

[B57-behavsci-15-01285] Rummel A.-M., Vogel A., Rosner R., Comtesse H. (2023). Grief-focused cognitive behavioral group therapy (G-CBT). Unpublished manual.

[B58-behavsci-15-01285] Semmlinger V., Ehring T. (2021). Predicting and preventing dropout in research, assessment and treatment with refugees. Clinical Psychology and Psychotherapy.

[B59-behavsci-15-01285] Smid G. E., Hengst S., de la Rie S. M., Bos J. B. A., Gersons B. P. R., Boelen P. A. (2021). Brief eclectic psychotherapy for traumatic grief (BEP-TG).

[B60-behavsci-15-01285] Smid G. E., Kleber R. J., de la Rie S. M., Bos J. B. A., Gersons B. P. R., Boelen P. A. (2015). Brief Eclectic Psychotherapy for Traumatic Grief (BEP-TG): Toward integrated treatment of symptoms related to traumatic loss. European Journal of Psychotraumatology.

[B61-behavsci-15-01285] Smith M. V. A., Grohmann D., Trivedi D. (2023). Use of social media in recruiting young people to mental health research: A scoping review. BMJ Open.

[B62-behavsci-15-01285] Tay A. K., Mung H. K., Badrudduza M., Balasundaram S., Azim D. F., Zaini N. A., Morgan K., Mohsin M., Silove D. (2020). Psychosocial mechanisms of change in symptoms of persistent complex bereavement disorder amongst refugees from Myanmar over the course of integrative adapt therapy. European Journal of Psychotraumatology.

[B63-behavsci-15-01285] UNHCR—The UN Refugee Agency (2024). Global report 2023—Executive summary.

[B64-behavsci-15-01285] Vogel A., Comtesse H., Nocon A., Kersting A., Rief W., Steil R., Rosner R. (2021). Feasibility of present-centered therapy for prolonged grief disorder: Results of a pilot study. Frontiers in Psychiatry.

[B65-behavsci-15-01285] Weathers F. W., Blake D. D., Schnurr P. P., Kaloupek D. G., Marx B. P., Keane T. M. (2013). The life events checklist for DSM-5 (LEC-5)—Standard.

[B66-behavsci-15-01285] Wiechers M., Strupf M., Bajbouj M., Böge K., Karnouk C., Goerigk S., Kamp-Becker I., Banaschewski T., Rapp M., Hasan A., Falkai P., Jobst-Heel A., Habel U., Stamm T., Heinz A., Hoell A., Burger M., Bunse T., Hoehne E., Padberg F. (2023). Empowerment group therapy for refugees with affective disorders: Results of a multicenter randomized controlled trial. European Psychiatry.

[B67-behavsci-15-01285] Wojtkowiak J., Lind J., Smid G. E. (2021). Ritual in therapy for prolonged grief: A scoping review of ritual elements in evidence-informed grief interventions. Frontiers in Psychiatry.

[B68-behavsci-15-01285] Znoj H.-J. (2004). Komplizierte trauer.

