# Supplementary material for: Treating Young Refugees with a Grief-Focused Group Therapy—A Feasibility Trial
Source: Behav Sci (Basel). 2025 Sep 19;15(9):1285. doi: 10.3390/bs15091285 (PMC12467290; doi:10.3390/bs15091285)
Supplement: Supplementary file 1 [file behavsci-15-01285-s001.zip › behavsci-3814745-supplementary.pdf]

What I am trying to avoid

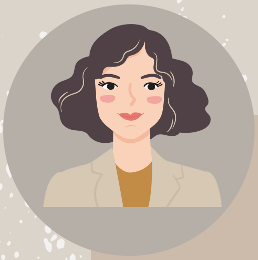

o Applies to me
o Does **not** apply

It looks like :______________________

____

What can I do?

- Writing a letter to the deceased:
- What do I miss most?

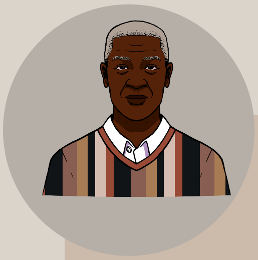

o Applies to me
o Does **not** apply

It looks like :______________________

____

What can I do?

- Looking at pictures and belongings of the deceased
- To face painful memories

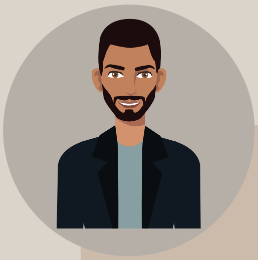

o Applies to me
o Does **not** apply

It looks like :______________________

____

What can I do?

- Writing about the event in detail (seen, thought, felt?)
- You can also write the story several times until it is correct.

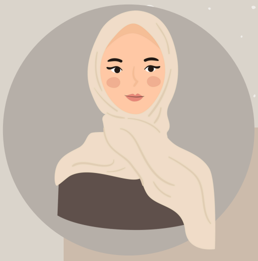

o Applies to me
o Does **not** apply

It looks like :______________________

____

What can I do?

- Reduce grief-related behavior
- Engage in positive activities instead

I will do that before the next session

____
